# Facilities that make the PDB data collection more powerful

**DOI:** 10.1002/pro.3788

**Published:** 2019-12-02

**Authors:** Joanna Lange, Coos Baakman, Arthur Pistorius, Elmar Krieger, Rob Hooft, Robbie P. Joosten, Gert Vriend

**Affiliations:** ^1^ Bio‐Prodict Nijmegen The Netherlands; ^2^ Centre for Molecular and Biomolecular Informatics (CMBI), Radboudumc Nijmegen The Netherlands; ^3^ Baco Institute of Protein Science (BIPS) Mindoro Philippines; ^4^ Department of Computer Science Dutch Techcentre for Life Sciences (DTL) Amsterdam The Netherlands; ^5^ Department of Computer Science Vrije Universiteit Amsterdam (VU) Amsterdam The Netherlands; ^6^ Biochemistry department Netherlands Cancer Institute (NKI) Amsterdam The Netherlands

**Keywords:** bioinformatics support, DSSP, PDB, protein structure bioinformatics

## Abstract

We describe a series of databases and tools that directly or indirectly support biomedical research on macromolecules, with focus on their applicability in protein structure bioinformatics research. DSSP, that determines secondary structures of proteins, has been updated to work well with extremely large structures in multiple formats. The PDBREPORT database that lists anomalies in protein structures has been remade to remove many small problems. These reports are now available as PDF‐formatted files with a computer‐readable summary. The VASE software has been added to analyze and visualize HSSP multiple sequence alignments for protein structures. The *Lists* collection of databases has been extended with a series of databases, most noticeably with a database that gives each protein structure a grade for usefulness in protein structure bioinformatics projects. The PDB‐REDO collection of reanalyzed and re‐refined protein structures that were solved by X‐ray crystallography has been improved by dealing better with sugar residues and with hydrogen bonds, and adding many missing surface loops. All academic software underlying these protein structure bioinformatics applications and databases are now publicly accessible, either directly from the authors or from the GitHub software repository.

## 1 INTRODUCTION

Macromolecular structure data are collected in the worldwide Protein Data Bank (wwPDB),^1^ currently at a rate of about 200 data entries per week. More than 2 million entries are downloaded daily by someone somewhere. This can, for example, be in an educational setting to learn more about one particular molecule of interest, to support drug design, or to analyze a genetic disorder. Protein structures are also used often in protein structure bioinformatics studies to learn more about the fundamentals of protein sequence–structure–function relations. The wwPDB associates a significant amount of metadata to each structure, and each of the wwPDB sites offers some web services to explore the entries. However, most of the software needed to study macromolecular structure in detail comes from other academic or commercial parties.

The wwPDB is a collaboration of three institutes together responsible for the maintenance of macromolecular structure data. All journals agree that deposition of macromolecular structure data is an obligatory step^2^ before an article describing that those data can be published, so that nearly all macromolecular structure data collected in academic research are publicly available. Anecdotal information suggests that the pharmaceutical industry has many more structures available than the 150K^3^ available from wwPDB, but these unpublished structures tend to be a large number of variants of an existing public PDB entry with different ligands bound.^1^

Although wwPDB is the primary source for 3D coordinate data, many analyses that are based on protein structures do not require going back to this primary source. A lot of analyses are based on a limited set of properties that can be derived from the 3D coordinates. Such analyses can benefit from the facility and consistency obtained by using databases of structure‐derived data. In the rest of this introduction, we will first explain in more detail why one would want to use derived structural parameters from a database, and then introduce some of the basic underlying facilities that we have developed for protein structure bioinformatics (PSB).

wwPDB provides an invaluable service in annotating entries, and curating their metadata, and hundreds of groups around the world built (and build) on their work to make secondary databases (i.e., databases that are computationally derived from the primary PDB database). Hundreds of articles have been written^4^ about secondary databases, but unfortunately many of these facilities no longer exist. We will discuss only facilities with a proven longevity. Good examples are the famous DSSP^5^ database and software by Kabsch and Sander, which have been maintained for the past 37 years; the WHAT IF^6^ web servers,^7^ which have been maintained for 21 years; and the HSSP^8^ multiple sequence alignments (MSAs) that have been kept up to date for 28 years already. The fact that these facilities have been cited thousands of times illustrates why it is worth the effort to keep them running.^9^

Before we address all other PDB‐wide secondary databases, web servers, web services, software, and information systems, we should start by highlighting the PDB‐REDO^10^ project. The PDB‐REDO database was released more than 10 years ago.^11^ Its aim is to improve the scientific quality and usability of the atom coordinates, occupancies, and B factors in PDB entries solved by X‐ray and electron diffraction, by re‐refining and rebuilding the structure model with today's software and a consistent protocol. A set of PDB‐REDO entries is often a better data source for structural studies than the corresponding PDB entries, as it reduces the effect of PDB structure models being made by different people, with different tools (and skills) at different times. Most CMBI's PDB facilities will migrate over the next 2 years from PDB to PDB‐REDO.

A nonexhaustive scan of the literature reveals that the users of the CMBI protein structure facilities include researchers in the fields of protein engineering, drug design, and structural biology, but most often in PSB. The facilities described in this article are especially useful for the latter group. All PSB software developers have at some point wasted their time writing code to parse PDB entries (e.g., http://emboss.sourceforge.net/apps/cvs/embassy/structure/pdbparse.html; http://biopython.org/wiki/Reading_large_PDB_files; http://biopython.org/DIST/docs/tutorial/Tutorial.html#htoc148; http://swift.cmbi.umcn.nl/teach/pdbad/). We produced Lists (http://swift.cmbi.umcn.nl/gv/lists/) to alleviate this problem for the aspiring PSB software developer. *Lists* consists of a series of databases, each of which contains one easy to parse entry with simple data per residue for each PDB entry. The “acc” database, for example, provides for each residue its accessible surface area, the “cys” database provides all cysteine bridges, the “chi” database provides all torsion angles, and so on. Presently *Lists* contains about 25 collections, but this can be extended upon request.

**Figure 1:**
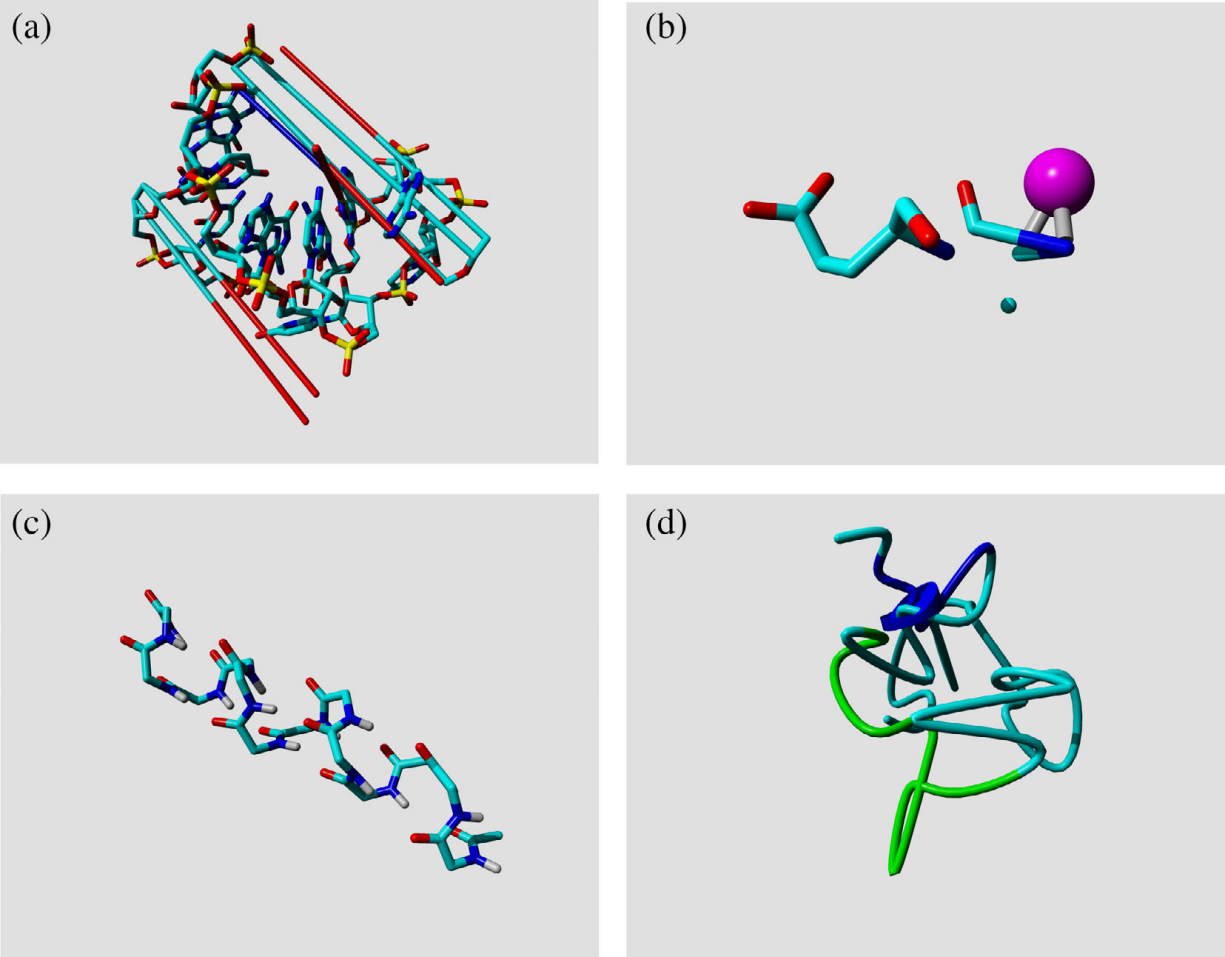
Examples that explain why not all PDB entries are equally useful for PSB studies. (a) The long lines indicate very long bonds in 1I4C. These seem to be caused by incorrect formatting of the PDB entry. Coordinates are written with only two characters before the decimal point, which makes them lose the minus sign when a coordinate is below −9.999; for example, an *X*‐coordinate like −11.236 becomes 11.236. (b) The *N*‐terminus of the azurin structure, 1AG0, once consisted of two half alanines with a copper ion in the middle. This problem has recently been solved, but the PDB provides no easy mechanism for correction tracking. (c) This small polyglycine helix (1CEK) is actually the result of a complex biophysical experiment. There is nothing wrong with this structure, but we believe that it should not be used in PDB‐wide computational studies. (d) Something went very wrong when solving this protein structure (2PDE, subunit‐binding domain of dihydrolipoamide acetyltransferase), the very short helix that is indicated by a short blue cylinder works as a chaotic attractor through which the chain passes seven times

The PDB contains more than enough entries to assemble a training data set and a test data set for most statistical (machine learning) studies. We can therefore afford to make a very strict selection. The CMBI PDB facilities include several tools to aid with this process. PDBREPORT holds for all PDB entries an extensive description of all anomalies, exceptions, errors, and things that our PDB parser did not understand. Figure 1 shows a few examples of PDB entries we believe are better not used in fully automated studies.

Table 1 gives a summary of all facilities mentioned in this article together with their main literature references and (very) brief descriptions. This table is available at the CMBI data facility page (http://swift.cmbi.umcn.nl/gv/facilities/) with an extensive explanation for each facility and with hyperlinks to those facilities.

**Table 1:** Summary of the facilities mentioned in this article

Facility	Short description
wwPDB^1^	Worldwide PDB. Macromolecular data collection and distribution.
UniProt^a^^,^ 12	Worldwide collection of protein sequences
Swiss‐Prot^b^	Manually annotated and reviewed section of the UniProt
PDB‐REDO^10^, ^13^ (improved)	Reanalyzed, consistently treated structure models
BDB^14^	PDB entries with standardized, isotropic B factors
DSSP^5^ (improved)	Secondary structure assignment for protein structure models
DSSP_REDO (novel)	Like DSSP, but for PDB‐REDO entries
WHAT IF^6^	Protein structure calculations. Outdated; some useful aspects as servers.
YASARA_View^c^	Free (feature‐rich) molecular viewer that additionally contains all of WHAT IF
HSSP^8^	Multiple sequence alignments against UniProt for proteins in PDB.
VASE^d^ (novel)	Visualization of entropy/variability values in HSSP entries
PDB‐Vis^e^ (novel)	Visualization of crystal packing contacts, and a few more things
LigPlot^f^	2D representation of ligand–macromolecule interactions
*Lists* (partly novel)	Sets of precalculated data for all PDB entries (see Table 3)
PDB_REPORT^15^ (improved)	Reports (in a PDF format) about anomalies and errors in PDB entries
WHY_NOT^g^	System that explains why certain data files are not present
pdbad^h^	Anecdotal list of problems in PDB entries
PDBFINDER	Easy to parse PDB metadata
PDBsum^i^	Summary of PDB metadata useful for PSB
LigPlot^j^	Visualizes interactions between macromolecules and ligands^16^
PDB_SELECT	Subsets of PDB entries (sequence unique at 30% cutoff)
PISCES^k^	More extensive system for making sequence unique subsets of PDB entries
CATH^l^, SCOP^m^, DALI^n^	Three facilities that shed light on the 3D relations between PDB entries
Swiss‐Model^o^	Builds homology models
Sternberg^p^ Brunak^q^	Examples of long‐time stable group pages with many useful facilities

*Notes*: The facilities in blue are produced by the authors of this article; the facilities in black are produced and maintained by others and we believe them to be useful for users of our facilities. Most of our facilities can be downloaded from ftp://ftp.cmbi.umcn.nl/pub/molbio/data/ and the software from the cmbi section in GitHub. YASARA_View can be downloaded freely from http://www.yasara.org/. http://swift.cmbi.umcn.nl/gv/facilities/ provides extensive documentation for the databases and instructions for obtaining an in‐house copy via rsync. Facilities without reference have not been published explicitly yet. References to some facilities are best extracted from those facilities' web pages. The facilities that are published here for the first time are labeled with the word “novel”. Facilities that underwent major updates since they were last published are labeled with the word “improved.”

a http://www.uniprot.org/.

b https://web.expasy.org/docs/swiss-prot_guideline.html.

c http://www.yasara.org/viewdl.htm.

d http://www.cmbi.umcn.nl/vase/.

e http://www.cmbi.umcn.nl/pdb-vis/.

f http://www.ebi.ac.uk/thornton-srv/software/LigPlus/.

g http://www.cmbi.umcn.nl/why_not2/.

h http://swift.cmbi.umcn.nl/teach/pdbad/.

i http://www.ebi.ac.uk/thornton-srv/databases/cgi-bin/pdbsum/GetPage.pl?pdbcode=index.html.

j https://www.ebi.ac.uk/thornton-srv/software/LIGPLOT/.

k http://dunbrack.fccc.edu/PISCES.php.

l https://www.cathdb.info/.

m http://scop.mrc-lmb.cam.ac.uk/.

n http://ekhidna2.biocenter.helsinki.fi/dali/.

o https://swissmodel.expasy.org/.

p http://www.sbg.bio.ic.ac.uk/.

q http://www.cbs.dtu.dk/services/.

## 2 DATABASES

Data collections, such as the PDBsum, DSSP, and so on, generally are referred to as databases. Even though some of these collections are database driven, most of them are a databank rather than a database (https://en.wikipedia.org/wiki/Data_bank). To not confuse the issue further, we will here keep making this same mistake and call each databank a database. In many cases, but not always, the name of the software and the name of the database are identical. Thus, the DSSP database is produced by the DSSP software, the HSSP database is produced by the HSSP software, but PDB_REPORT and *Lists*, for example, are produced by WHAT IF.

### 2.1 *PDB‐REDO reanalyzed and re‐refined PDB entries solved by crystallography*

**Figure 2:**
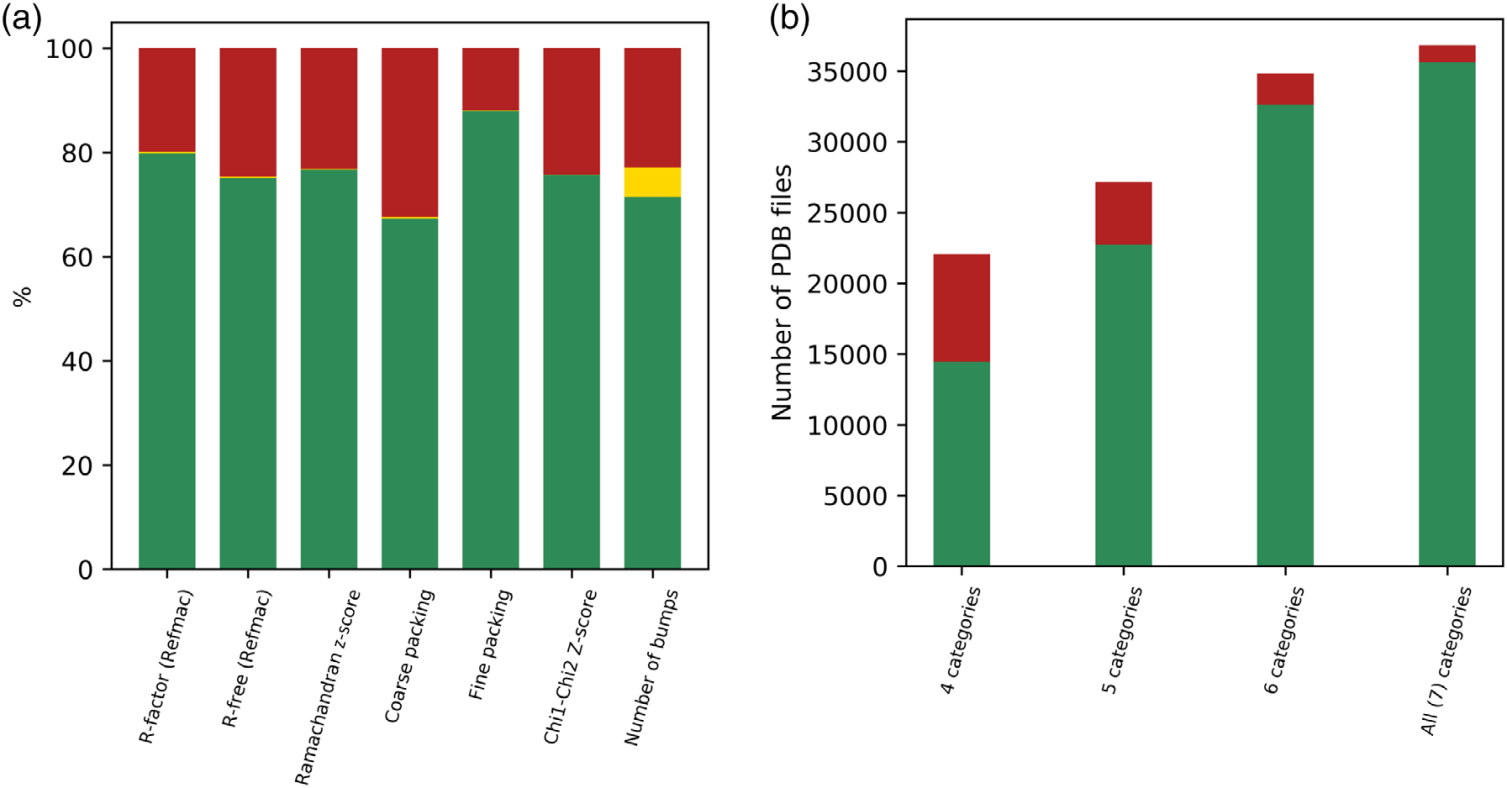
Protein structure quality improvements obtained by PDB‐REDO. (a) Percentage of files for which the PDB‐REDO entry showed “better” characteristics in green, PDB better in red, and equally good in yellow. (b) This shows how often the PDB‐REDO entry was better/worse than the PDB entry for 4 to 7 of the categories used in (a). These analyses were run on all entries available in August 2019. We manually checked many of the 1,195 cases for which all seven categories showed worse statistics for the PDB‐REDO entry and found many examples of structures solved at extremely low resolution (like 6.0 A or lower). We also observed that the distribution of cases in which all model quality indicators deteriorated was skewed toward being more recently made PDB‐REDO entries. This was (at least partially) traced back to bugs in third‐party programs, which PDB‐REDO did not intercept correctly. These bugs are being fixed, and the affected PDB‐REDO entries will be replaced

PDB‐REDO uses the latest crystallographic software from the developers and third parties and today's CPU power to improve the way atomic coordinates (and occupancies and B‐factors) reflect the underlying experimental data (the reflections that were experimentally determined). Figure 2 shows that the vast majority of PDB entries can be improved both in terms of crystallographic quality statistics and in terms of protein structure “normality”. The recent efforts in PDB‐REDO have focused on making models more complete^13^ and making sets of homologous protein structures more consistent to allow focus on the actual differences between homologs.^13^ The fact that most PDB‐REDO entries have better crystallographic quality parameters (*R*, *R*_free_) than their PDB originals indicates that PDB‐REDO entries indeed provide a better representation of the underlying reflection data than the corresponding PDB entries. In terms of model completeness, more than 95% of the side chains that are (partly) missing in the PDB were modeled by PDB‐REDO based on the prior knowledge that they are there in the structure and that they have a limited set of possible conformers (rotamers) and on the experimental data that, albeit sometimes with only very weak signal, gives an indication where the side chain resides. Additionally, almost 20K missing loops were added. The potential of adding more than 16K missing carbohydrates (such as fucose and mannose residues) was recently shown,^17^ and these are now added gradually by replacing PDB‐REDO entries.

### 2.2 *PDBREPORT lists anomalies in PDB entries*

PDB entries are the result of a long process that starts with purification of the macromolecule for biophysical experiments and ends with structure deposition in wwPDB. The steps in‐between all come with their own systematic and random experimental errors. Consequently, PDB entries will not be error‐free. In 1993, the first three structure validation methods were published in rapid succession: Directional Atomic Contact Analysis (DACA),^18^ PROCHECK,^19^ and Protein Structure Analysis (ProSA).^20^ These three methods determined rules from protein structures solved at high resolution—that are therefore presumed “correct”—to find anomalies and errors in protein structures in general. Hundreds of structure validation methods have been published since 1993, and the WHAT_CHECK^15^ software has about 200 modules that each check another aspect of a PDB entry. The full WHAT_CHECK reports are available from the CMBI facilities site as human‐readable PDFs.

### 2.3 *DSSP secondary structure determination*

DSSP is the oldest PDB‐derived database. Conceived in the late 1970s, only a few years after the PDB itself, it was first published in 1983.^21^ Around 1995, the software was rewritten completely by Michael Scharf in the Sander group at the European Molecular Biology Laboratory (EMBL) in Heidelberg. Around 2009, the software was rewritten again by Maarten Hekkelman at the CMBI in Nijmegen, this time including the assignment of π‐helices. This latter version is available on GitHub, and it is also distributed by CCP4 (https://www.ccp4.ac.uk/). DSSP has many different applications: it is used for improving sequence alignments,^22^ validating the quality/normality of protein structures,^19^, ^23^ analyzing molecular dynamics trajectories,^24^ homology modeling,^25^ protein structure comparison,^26^ protein structure classification,^27^ analyzing disease‐causing human mutations,^28^ solving structures by NMR,^29^ genome annotation,^30^ mapping life, the protein universe and everything,^31^ and thousands of secondary structure prediction software design experiments.^32^ Consequently, the DSSP article has been cited more than 10,000 times. DSSP is hardly ever used to directly answer a biological question, except perhaps for the design of thermolabile mutations or peptidic epitope mimetics. Indeed, most of the citations to the DSSP article are found in Methods papers that are either of a fundamental nature or describe methods and servers that can help life science researchers with their work. DSSP is such a basic, and important, protein structure analysis facility that we can imagine that wwPDB would benefit from incorporating the latest version in their software suite.

**Figure 3:**
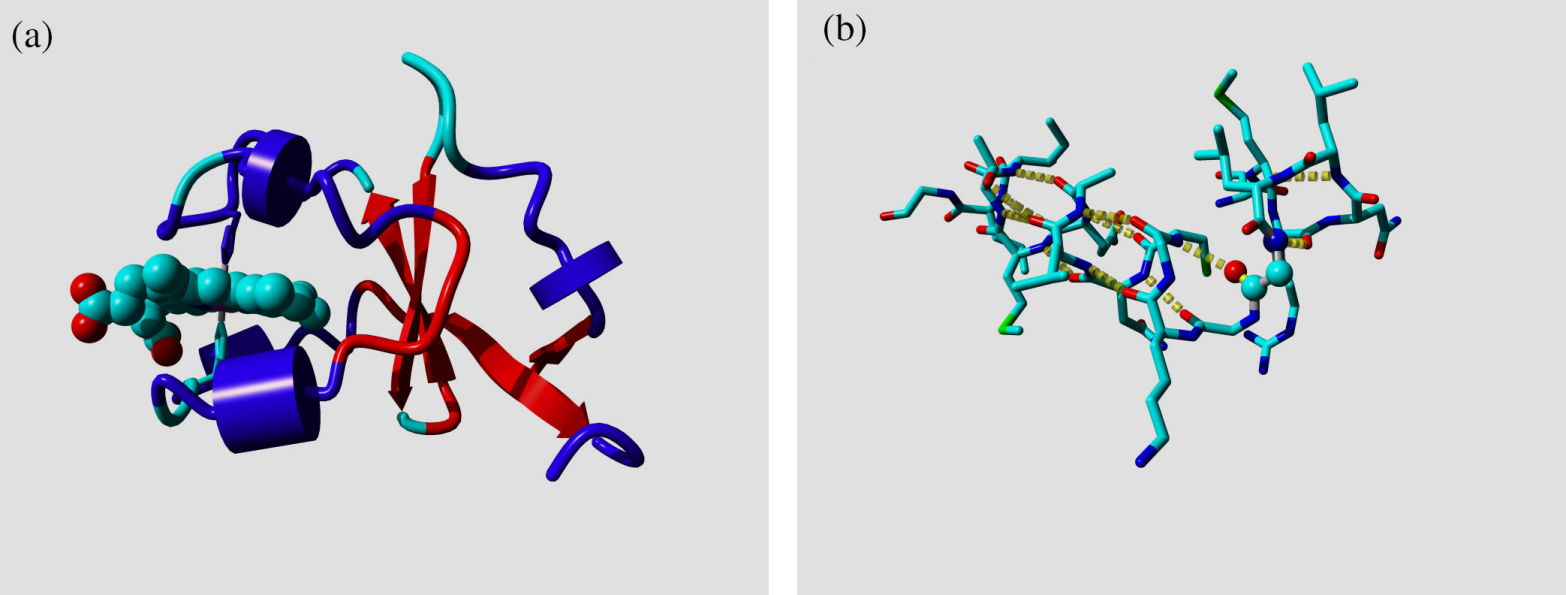
Examples of secondary structure assignments in PDB entries. (a) Ribbon drawing of http://firstglance.jmol.org/fg.htm?mol=1M2I (Cytochrome b5) colored by the PDB‐assigned secondary structure. Cyan: not assigned; blue: helix; red: sheet. The ball model is a heme group that is held by two histidine side chains (shown in cyan and blue). The agreement in secondary structure assignment between YASARA and DSSP is higher than that between PDB and DSSP. Additionally, one 3_10_‐helix is called helix in the PDB entry, while another 3_10_‐helix is not assigned. WHAT IF normally reduces the eight‐character DSSP alphabet to a four‐character one in which 3/10‐helix and π‐helix are combined with α‐helix into H, and nonhydrogen‐bonded strands are called loop. (b) Example of a nearly unsolvable problem. The glycine (Gly‐20) in http://firstglance.jmol.org/fg.htm?mol=1KC7 (pyruvate phosphate dikinase) that is located in the corner “between” the longer horizontal helix and the single‐turn helix in the upper right actually has a H‐bond in each of these two helices. This makes it look like 1 long helix in DSSP and in Lists entries. The only solution we see for this rare problem (glycine 107 in http://firstglance.jmol.org/fg.htm?mol=1L51, lysozyme is another example) is manual annotation in PDB entries. Most programs, including DSSP and YASARA, have their own way of dealing with secondary structure assignments often based on hydrogen bond assignments with cutoffs that are a bit arbitrary. It seems wise to always use the same arbitrariness. That is why we decided to make DSSP open source with a very permissive license so that DSSP can be incorporated in other programs

The classic PDB format of the wwPDB contains a secondary structure assignment; however, this information is not as robust as the information from DSSP. wwPDB is concentrating its efforts on the modern mmCIF serialization of their data entries, but many PSB users are still using the PDB‐formatted files. It is therefore worrying that we found several types of errors in the embedded secondary structure assignments. The most striking is the fact that PDB greatly overassigns helices; http://firstglance.jmol.org/fg.htm?mol=1KEZ (macrocycle‐forming thioesterase domain), for example, has 165 residues (20% of all residues) incorrectly assigned as helix in the PDB entry. Several entries have only α‐helix assignments but no β‐strand assignments; even when the structure consists largely of β‐strands like http://firstglance.jmol.org/fg.htm?mol=4PCH (Human Polyomavirus 7 [HPyV7] VP1 pentamer), in which the PDB entry has not assigned any β‐strand while according to DSSP there are 569 β‐strand residues. Apart from assignments being different between PDB and DSSP, we also encountered many administrative mistakes in the PDB assignments. Examples are secondary structure assignments that spread over multiple chains, for example, http://firstglance.jmol.org/fg.htm?mol=1H1L (nitrogenase MoFe protein) and http://firstglance.jmol.org/fg.htm?mol=1E6Y (methyl coenzyme M reductase) or assignments for residues that are not present in the PDB entry (e.g., http://firstglance.jmol.org/fg.htm?mol=1E6Y). We could not find TURN records in any PDB entry, except for a few turns that were annotated on HELIX records. Many PDB entries contain duplicated secondary structure records. Figure 3 shows a typical case of DSSP versus PDB differences. Many of these problems have been solved with the introduction of mmCIF files, but aspiring protein structure bioinformaticians tend to at least start their project with PDB‐formatted files as these can be read by a human.

DSSP entries should be used instead of the PDB annotations if a consistent secondary structure assignment is required. wwPDB might consider removing most secondary structure assignments from their data entries, leaving in only those annotations that are required to express important facts observed by the scientist who solved the structure. DSSP assignments also have their peculiarities, but these are known and systematic, which for a systematic analysis in PSB should be preferred over random and erratic. Table 2 illustrates the inconsistency between PDB and DSSP secondary structure assignments.

**Table 2:** Secondary structure assignments of PDB versus PDB‐REDO

*N*‐term	REDO longer	REDO shorter
1	1,500	1,298
2	660	268
3	303	310
>3	230	87
***C*‐term**	**Longer**	**Shorter**
1	9,761	6,368
2	2,256	1,106
3	637	459
>3	531	220

*Notes*: The DSSP assignments were compared of 135K helices that were at least six residues long in the PDB‐REDO entry. We then asked how often the helix in PDB‐REDO was shorter or longer than in the corresponding PDB entry. In 1500 cases, the helix in the PDB‐REDO entry was one residue longer than in the corresponding PDB entry. Re‐refinement by PDB‐REDO tends to make helices more often longer than shorter. 4% of the residues assigned differently near helix ends are assigned strand in one of the two files. 108K helices were equally long in the two corresponding entries.

### 2.4 *DSSP_REDO holds DSSP entries for all PDB‐REDO entries*

DSSP_REDO holds DSSP entries for all PDB‐REDO entries similar to the DSSP database holding DSSP entries for native PDB entries (that contain protein data). When comparing 135K helices between the PDB entry and the corresponding PDB‐REDO entry, we observe that 20% of these pairs have length differences (see Table 2). The fact that length differences are much more frequent at the C‐terminal end than at the N‐terminal end can be easily explained from geometrical characteristics of helices in general, but that is beyond the scope of this article. Secondary structure determination software generally depends on the determination of hydrogen bonds that in turn depend on a series of (arbitrarily set) cutoff parameters. So, if the distance between a backbone nitrogen and oxygen is, for example, 3.51 Å in a PDB entry and 3.49 Å in a PDB‐REDO entry, then this might lead to an assignment difference of one position between these two databases. It should be noted that PDB‐REDO uses (homology‐based) hydrogen bond restraints at lower resolutions,^13^ which may be a reason for the observed changes in secondary structure. Human inspection of a few dozen examples, though, seems to suggest that the DSSP‐REDO based secondary structure assignments often are better for PDB entries than for the DSSP assignments from those PDB entries themselves.

### 2.5 *HSSP entries contain multiple sequence alignments for proteins in PDB entries*

**Figure 4:**
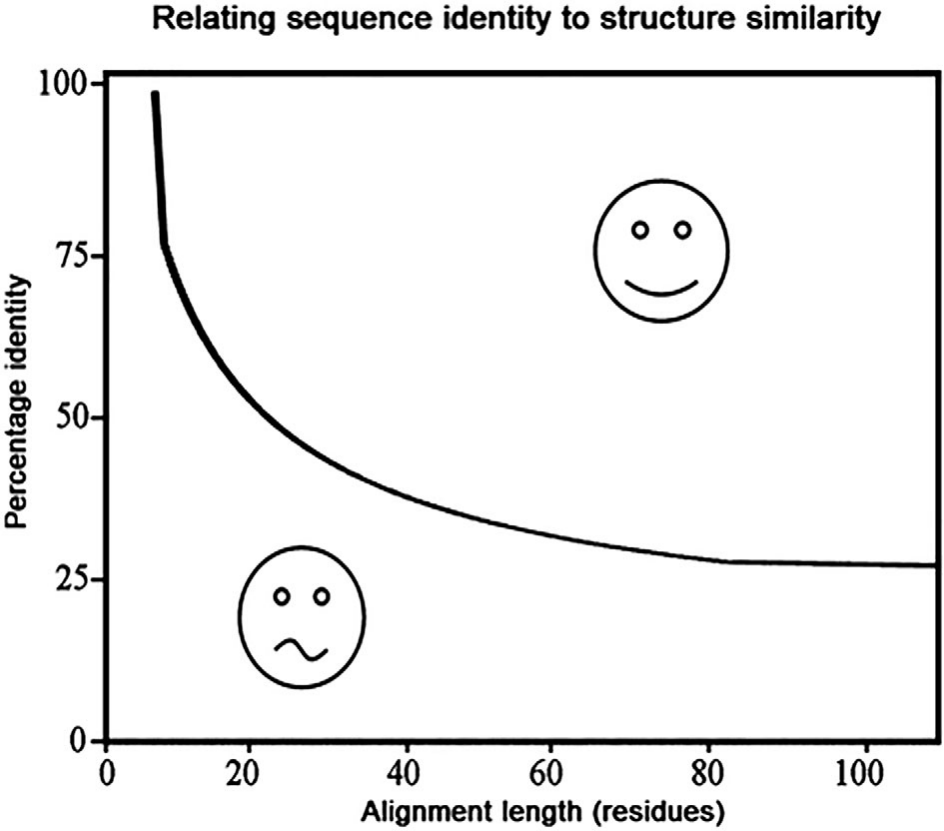
Homology modeling threshold curve. This plot describes which minimum percentage sequence identity in an alignment of a given length is an indication that the aligned proteins have similar structures. This is frequently used in the context of homology modeling: alignments above the curve indicate that it is possible to make a fairly reliable homology model from the aligned template; alignments below the curve mean that a homology model should be handled with care. We also use this plot for the inclusion of sequences in HSSP alignments

The HSSP facility couples the protein structure database with homologous sequences from the protein sequence database. One of the main concepts in bioinformatics is that information can be carried over between homologous proteins. If two proteins have a high enough gene sequence similarity to infer that they are homologs, then information about the function of one protein can be carried over to the other one. The same holds for the individual amino acids: if amino acids line up in an MSA, then biomedical information obtained for one residue can be carried over to the equivalent (aligned) residues in the other sequences. The level of certainty of this carrying over of information is as high as the confidence that the residues actually should line up in the MSA. This confidence is higher when the proteins have evolutionarily diverged less or when the alignment can be derived from superposed protein structures.^33^ The HSSP^8^ databank holds for every protein sequence in the PDB an MSA that is produced in an iterative one‐protein‐centered way. The HSSP production process has been described extensively.^8^ In short, BLAST is used to find homologs of the protein chain for which the alignment is produced. In the first round, similar sequences are aligned to the one protein of interest. From this alignment, a sequence profile is produced and this profile is used to align more sequences that per iteration are allowed to be less similar, this new alignment is then used to produce a new profile, and so on. This iterative process stops when no new sequences are found above a threshold curve (see Figure 4). Rost later made improvements to this curve.^34^ The CMBI facilities use the original curve to which 5% sequence identity is added over the full length. The threshold curve in Figure 4 levels off at 25% so that no HSSP entry will hold a sequence with less than 30% sequence identity to the sequence for which that HSSP entry was produced. Additionally, all aligned sequences match over 75% of the full length.

### 2.6 *The XSSP server produces HSSP and DSSP files on demand*

HSSP entries tend to be recalculated once every 2 years to widen their coverage. The XSSP server at http://www.cmbi.umcn.nl/xssp/ will take a valid PDB entry or the 4‐letter PDB id (for PDB or PDB‐REDO entries) and produce a fresh HSSP entry based on the UniProt contents of that moment. Producing an HSSP entry is a burden to our compute facilities so we hope that people who need large numbers of “fresh” HSSP entries will install an in‐house version of the software, which is called XSSP and can also produce DSSP entries. XSSP can be obtained from GitHub: https://github.com/cmbi/xssp. The HSSP entries can be obtained in two formats: Native HSSP^8^ and Stockholm^Q16^ format.

### 2.7 *The PDBFINDERs summarize PDB entry metadata in a standard way*

**Figure 5:**
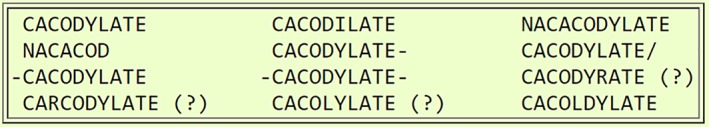
Some variants of the word cacodylate found in PDB entries. The question marks indicate that those words were also found a few dozen times in the literature, at locations where cacodylate could be expected

PDB entries are notoriously hard to parse. For example, there are more than a hundred different ways to describe a phosphate buffer, nearly a hundred ways to describe a tartrate buffer, and even the simple word cacodylate knows many variants (see Figure 5). We see a challenge for the machine‐learning community.

**Figure 6:**
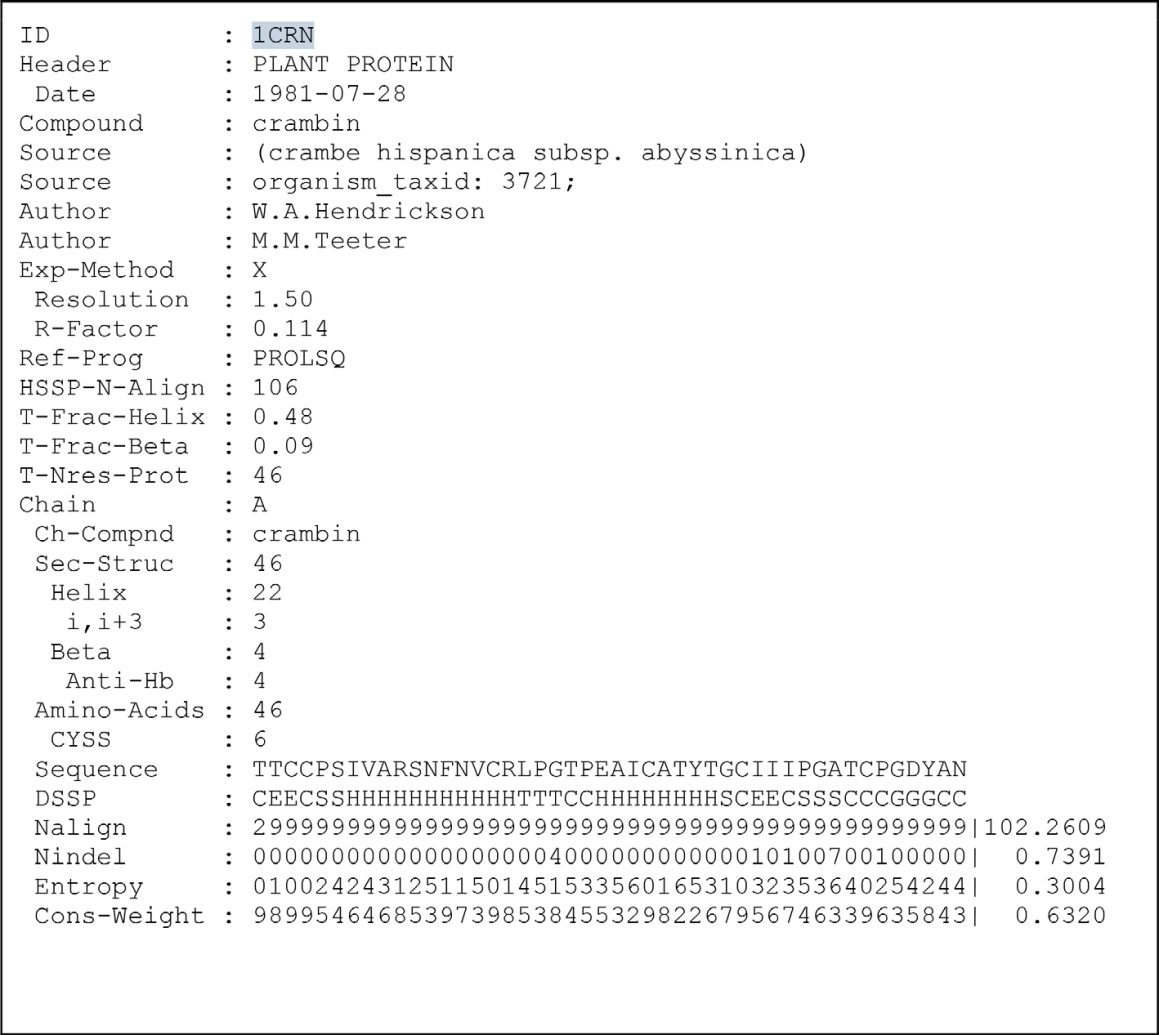
PDBFINDER entry for the PDB entry 1CRN (crambin). Most key value combinations are self‐explanatory. Indentation of a key indicates that it is a child of the unindented parent above it. The six bottom lines are extracted from the corresponding DSSP and HSSP entries. For the bottom four lines, the HSSP‐derived values are scaled to 0.0–9.0 and represented by the nearest integer to that scaled number. PDBFINDER2 entries additionally hold many lines of calculated per‐residue information, including average B factors, packing normality, geometric anomalies, side chain flips, and so on

We produced the PDBFINDER to ease the burden of parsing PDB headers. PDBFINDER holds for each PDB entry a small summary of the metadata we believe are essential for PSB. Figure 6 shows the PDBFINDER entry for PDB entry 1CRN. Note that this entry holds no information about the crystallization conditions as those are typically too incomplete and/or unstructured in PDB entries to extract useful information from. If the PDB format had been designed last year instead of that in the 1970s, it most certainly would have looked very, very different, and indeed wwPDB is threatening already for several years to move entirely to the more modern (but still not very pleasant to parse) mmCIF format (https://www.wwpdb.org/news/news?year=2019). Files that are too big for the PDB format (e.g., more than 99,999 atoms) are available only in the mmCIF format. For these we do produce DSSP entries and PDB‐REDO entries, but other facilities, today, are still produced from PDB‐formatted files only.

The parser that combines PDB, DSSP, and HSSP into PDBFINDER is the result of generation upon generation of programmers who dealt with one idiosyncrasy after the other. For example, we once observed an R‐factor value written out as “seventeen” in characters rather than digits and modified the PDBFINDER software to deal with this properly. The PDBFINDER scripts also cope, for example, with uncommon descriptions of experimental techniques, many permutations of describing R‐factors, the date of the file (which is not always given on Line 1), EC‐code (which sometimes does not exist), and the many variants (and combinations of) refinement programs. In the early 1990s, we once looked up in the literature all nearly 200 missing R factors and made those available to the PDB at Brookhaven. At that time, we also reported thousands of other inconveniences. Many people have done similar things over the years so that the number of parsing problems slowly decreases even though the size of the PDB is increasing. Several PDBFINDER parser modules probably have been obsoleted by wwPDB patches and remediations.

PDBFINDER2 entries are largely identical to PDBFINDER entries, but, when possible, contain extra lines that encode for each amino acid computationally derived data such as its accessibility, B‐factor, geometric anomalies, and a series of normality scores extracted from the PDBREPORTs. Actually, MRS returns the PDBFINDER2 entry even when the PDBFINDER entry is requested, as all lines are always identical up to the extra lines. The quality indicators in PDBFINDER2 are mainly meant to support homology modeling projects (e.g., HOPE^33^ or YASARA_View^35^), and seem less useful for human inspection. The PDBFINDERs are mainly meant for use in software, albeit that they are human readable too. For casual inspection of the metadata of an individual PDB entry, we recommend using the PDBsum^36^ system.

### 2.8 *Lists databases provide easy to parse data that is computationally derived from PDB entries*

We also provide facilities that allow people to avoid parsing the PDB entries altogether. Many PSB software design projects require only little information from the PDB entry. For example, secondary structure prediction projects normally require a training data set and a test data set that are much more easily obtained from DSSP entries than from PDB entries. We produced a large number of databases that can help protein structure bioinformaticians doing their work without the need to write or import their own PDB parser. We produced, for example, databases with all torsion angles in proteins, accessibility values, all τ angles, and so on. We call these the *Lists* databases. Table 3 summarizes the *Lists* databases available at the time of writing. Each *Lists* database has a three‐letter name, like “chi” for the database that provides the torsion angles for all amino acids in all PDB entries, “acc” for the database with solvent accessibility values, “cnu” for amino acid–nucleotide interactions, and so on.

**Table 3:** Available Lists databases

*Lists* name	Derived data type
chi	Torsion angles (φ, ψ, Ω, χ1‐5)
tau	Backbone angle τ
acc	Accessible molecular surface area
asa	Accessible surface area
dsp	Secondary structure overview
cc1	Cα–Cα distance <12.5 Å
cc2	Residue spheres closer to 0.25 Å
cc3	Residue spheres closer to 2.5 Å
cc4	Residues with an atom pair closer to 0.25 Å
cc5	Residues with an atom pair closer to 2.5 Å
cc6	Residues with a side chain atom pair closer to 0.25 Å
cc7	Residues with a side chain atom pair closer to 2.5 Å
cc8	Residues in different chains with a side chain atom pair closer to 2.5 Å
cc9	Cβ–Cβ distance <12.5 Å
cli	Residues with an atomic contact to ligand <1.0 Å
cnu	Residue‐nucleic acid spheres closer to 1.0 Å
iod	Residue distance to the nearest (positive) ion
ion	Short residue–ion distances, grouped per ion
cys	Cysteine bridges
sbr	Salt bridges
sbh	Salt bridges assuming histidine is positive
qua	Coarse‐packing quality
nqa	Fine‐packing quality
flp	Backbone peptide‐plane flips
rot	Residue rotamer scores
sou	WHAT IF's interpretation of the PDB entry
sco	Gives each PDB entry a bioinformatics–usability score from 0.0 to 10.0

*Notes*: Each Lists database is extensively described at the Lists website. Angles, for example, in the “chi” and “tau” databases are in degrees between −180.0 and 180.0; accessibility values in “acc” and “asa” are in square Ångströms, missing values often are set at −999.9, and so on. These Lists databases are grouped. Databases that provide elementary geometric parameters are listed in red. Amino acid contact databases intended for use in protein structure prediction PSB projects are in blue. Databases with other contacts are in green. Databases related to protein structure quality and normality are in yellow. The “sou” and “sco” databases, in purple, are special and are explained in the text.

The criteria for entering a PDB entry into *Lists* are more stringent than for the other databases because when doing bioinformatics studies we believe one should neither include small peptides, nor structures solved at low resolution, nor structures that contain very many ligands/co‐factors, nor ribosomal or viral coat proteins as these are hard to study in their complete 3D environment. For each file that did not pass the acceptance criteria a small file with the extension “.whynot” is written that explains why the file was not produced. These “.whynot” entries propagate to the WHY_NOT system that is available for human inspection through the WHY_NOT server.

**Figure 7:**
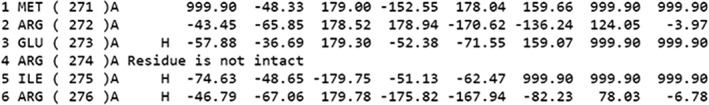
First few lines of an example chi Lists database. Entry: From left to right, the columns are the sequential residue number; the residue type; the PDB residue number; the protein chain identifier; the secondary structure according to DSSP (in a reduced alphabet); φ, ψ, Ω, and χ1‐5. No calculations are done for residues that are not completely intact. The text “Residue is not intact” is used for this purpose throughout all Lists databases

Table 3 shows which *Lists* databases are present. Most are self‐explanatory and all are explained extensively at the website (http://swift.cmbi.umcn.nl/gv/lists/). The *Lists* database “sou” gives for each PDB entry a summary of the WHAT IF interpretation of that entry. This database is needed because it is impossible to check all entries by hand, and crystallographers keep solving all the time more complicated structures with scientifically more complicated exceptions that WHAT IF has never seen before so that the occasional parsing error seems likely. In such cases, the user can check the corresponding “sou”‐entry (and hopefully warn us if WHAT IF makes a parsing error). Figure 7 shows, as an example, the top six lines of the “chi” database entry for the PDB entry 1CR1.

The *Lists* database “sco” gives every PDB‐REDO entry a bioinformatics usability score from 0.0 to 10.0. These scores follow the Dutch school exam grading system in which 10.0 is perfect, 6.0 is just a pass, 5.0 is doubtful, and 4.9 and below is bad. Be aware, though, that these scores definitely are neither a quality judgment for the PDB entry nor for the experimentalist; these scores merely indicate how useful an entry is for a protein structure bioinformatician who needs a training set and a test set to perform a study on “normal” protein structures. The “sco” scoring system starts by giving each entry 10.0 points and then subtracts points when the resolution is worse than 3.0 Å; when the longest contiguous protein chain in the entry is shorter than 50 amino acids; when the entry contains UNK residues, Cα‐only residues, or residues with otherwise missing atoms; or when the entry holds residues or ligands that WHAT IF cannot interpret because it does not have an entry for it in its topology database. When after subtracting all these administrative penalty points the score is still positive, the Ramachandran score^23^ and the DACA packing score^18^ are determined and added to the score. At the end, scores are truncated at the interval 0.0–10.0.

### 2.9 *The WHY_NOT system/database explains why database entries are missing*

Many database entries either make no sense or cannot be produced. DSSP entries for PDB entries of RNA structures, for example, make no sense, and DSSP entries cannot be produced if the PDB entry holds only the coordinates for the Cα atoms of the protein(s). PDB‐REDO entries cannot be produced if the underlying reflection data are not available. Cysteine bridge *Lists* entries are meaningless for a structure without cysteine bridges, and so on. The WHY_NOT system holds track of all missing database entries and can tell the user why missing database entries actually are missing. For some databases, WHY_NOT has only a few entries and knows only a few entry types, while for others, the number of WHY_NOT entries make up more than half of the database. Table 4 provides a few examples.

**Table 4:** The “acc” Lists database has no data entry (and thus a .whynot entry) for more than 30K PDB entries

11,027	COMMENT: MODEL records found
3,064	COMMENT: Not an X‐ray structure
102	COMMENT: Not enough intact residues
1950	COMMENT: Not enough residues
438	COMMENT: Percentage bad residues too high
15,729	COMMENT: Too many bad residues
440	COMMENT: Too many C‐alpha‐only residues
825	COMMENT: Too many residues

*Notes*: 11K of those are either structures solved by NMR or multimodel X‐ray files; more than 15K entries are missing because they hold too many amino acids with missing atoms (mainly side chain atoms of Glu, Arg, Lys, and Gln); 3K are missing because they are solved by another technique compared with X‐ray technique (these are mainly EM structures, and structures solved by NMR for which only one structure is given rather than a multimodel ensemble). Other Lists databases know other entry types. For example, the Lists database for cysteine bridges has about 24K entries “COMMENT: Contains no cysteine bridges” and about 7K entries “COMMENT: Contains no cysteines”. These criteria are applied first for the “cys” database, and therefore the entry “COMMENT: Too many bad residues” occurs only a few thousand times in the “cys” database rather than almost 16K times for the “acc” database.

## 3 SOFTWARE

### 3.1 *The WHAT IF general‐purpose macromolecular visualizer has been succeeded by YASARA*

**Figure 8:**
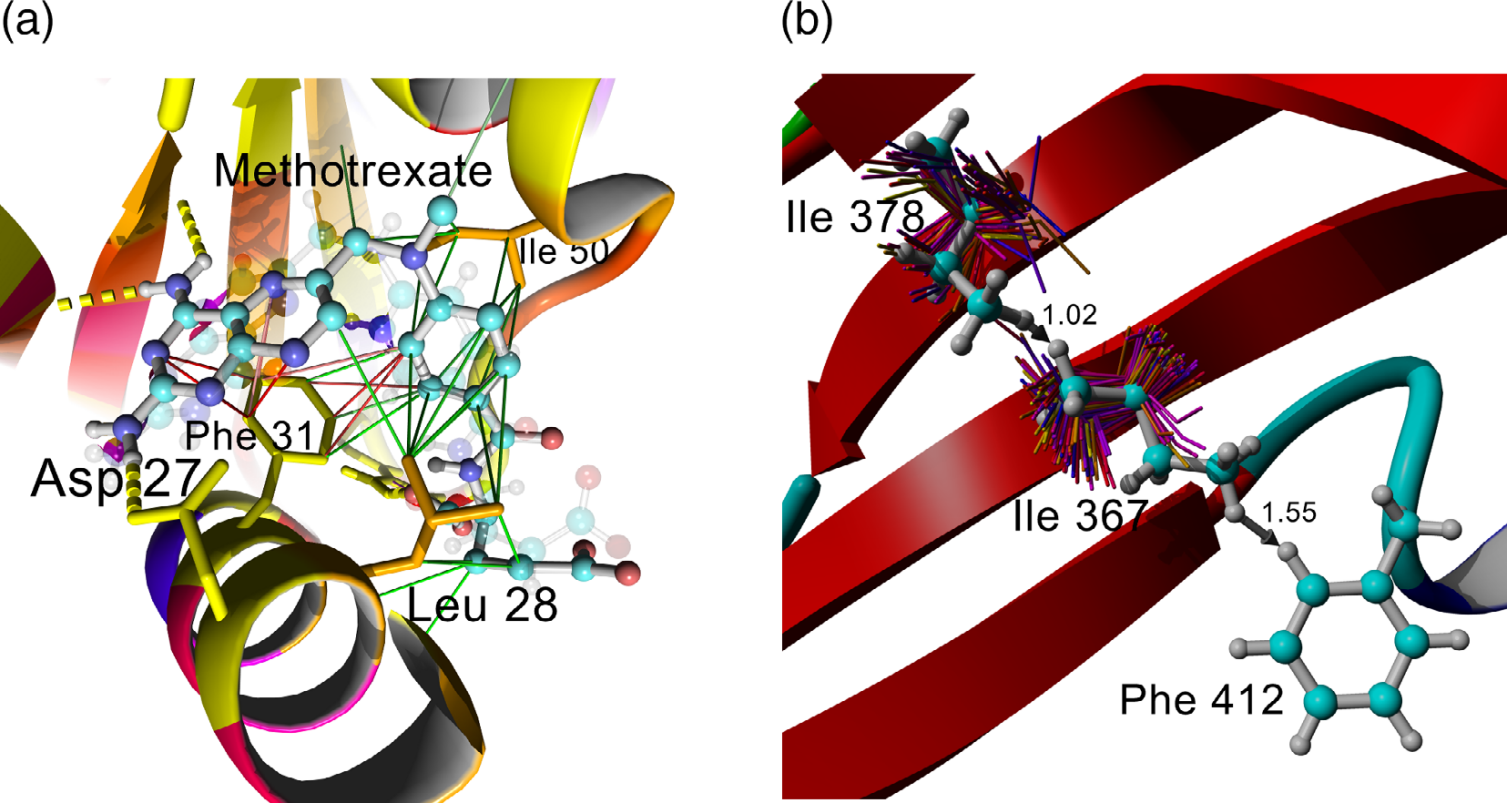
Examples of WHAT IF calculations visualized in YASARA_View. (a) Crystal structure of dihydrofolate reductase with inhibitor methotrexate (**4DFR**). Protein colored by HSSP conservation weights found in the PDBFINDER2 database from blue (not conserved) via red to yellow (totally conserved). Contacts between protein and ligand are shown: hydrogen bonds (yellow), hydrophobic contacts (green), and π‐interactions (red). (b) Crystal structure of the protein TolA domain III (**3QDR**). Strong clashes are reported by the PDBREPORT and PDBFINDER2 databases for residues Ile 378, Ile 367, and Phe 412 (indicated with gray arrows). Backbone‐dependent Ile rotamers from the WHAT IF DGROTA option are shown as thin sticks and colored from blue to yellow. Clashes can be resolved in this example by choosing more populated rotamers

Work on WHAT IF started December 7, 1987, and has never stopped. However, WHAT IF is now long past its expiration date, and it is only distributed when really needed. Furthermore, the stable version of WHAT IF 2006 is freely available inside the YASARA software, also in the free‐of‐cost YASARA_View version (http://www.yasara.org/viewdl.htm and https://en.wikipedia.org/wiki/YASARA) that was specially designed for education purposes. Figure 8 shows a few examples of WHAT IF results visualized through YASARA_View. Many WHAT IF options are still unique and useful for a wide variety of PSB projects, including the CMBI HOPE server. These options are made available as a web server and/or a web service. The WHAT IF web servers are available at http://swift.cmbi.umcn.nl/ and the web services at http://wiws.cmbi.umcn.nl/wsdl/ and http://wiws.cmbi.umcn.nl/help/.

### 3.2 *The MRS data retrieval system gives easy, rapid access to many databases*

The MRS data retrieval system^37^ provides rapid access to a large number of databases, including PDB, PDB‐REDO, HSSP, DSSP, the PDBFINDERs, and PDBREPORT. Entries retrieved by MRS as the result of a user query are extensively hyperlinked to other databases, both inside and outside MRS. The MRS user interface includes a bit of artificial intelligence that sorts the hits it believes are most relevant to the user to the top of the hit list. For example, a search for “crambin” in the PDB sorts crambin structures solved by X‐ray to the top of the list followed by structures solved by NMR or neutron diffraction and structures that contain a crambin fold or hold a reference in which crambin is mentioned. MRS is one of the core components of the eBioKit,^38^ which plays a crucial role in the development of bioinformatics facilities and skills in Africa. MRS is freely available from https://github.com/cmbi/mrs.

### 3.3 *VASE allows for visual inspection of HSSP multiple sequence alignments*

HSSP entries not only hold multiple sequence alignments but also additional data like the variability and entropy per sequence position. The VASE software displays in one window the HSSP MSA; the variability, entropy, and weight per position, extracted from the HSSP entry; and a small JMOL picture that indicates the position in 3D of residues selected by the user in the other two representations. VASE is not a spectacular application that provides mind‐boggling new insights, but more a starter package for researchers who want to “attach” an interactive website to an article about a protein sequence–structure–anything study. VASE can be obtained from GitHub: https://github.com/cmbi/vase. Vase automatically creates an HSSP entry from an input structure model by running XSSP under the hood.

### 3.4 *PDB‐Vis gives interactive access to crystal packing analyses*

X‐ray crystallography starts with growing crystals.^39^, ^40^ Proteins pack upon each other in a very regular way to form these crystals. This packing is non‐natural, and there where the proteins pack, artificial situations arise that can lead to misinterpretations of many aspects of the protein. Examples are ions that seem bound by only two liganding groups when the other two ion‐binding residues are located in a symmetry‐related molecule. Many good molecular structure visualization tools exist, and most of these visualizers provide much useful functionality. Crystal contacts, though, tend to be provided only by visualizers that were written for X‐ray crystallographers and tend to be missing in visualizers written for drug design or protein engineering purposes. We have therefore produced PDB‐Vis, a server that takes a 4‐letter PDB identifier, as input and produces a plot in which the user can see which residues make symmetry contacts. This server also produces a scene for YASARA_View in which each residue is colored by its number of symmetry contacts. One of the *Lists* databases holds for every PDB entry a copy with a shell of symmetry‐related residues included; not many browsers can deal with these files though because too many residues are present with the same name, chain, and number. PDB‐Vis can also visualize contacts of the macromolecule with ions and ligands. We suggest using LigPlot^16^ for visual inspection of ligand contacts in 2D.

## 4 HUMAN GENOME‐RELATED PROTEIN STRUCTURE FACILITIES

### 4.1 *HOPE analyzes disease‐causing mutations in human proteins*

Nature genetics published an editorial in 2012 in which they state that it will no longer be enough to find out which point mutation is causally related to a genetic disorder and urge the human genetics researchers to also find out why that point mutation is causal for the disease state.^41^ When the 3D structure of the (mutated) protein—or a reliable homology model thereof—is available, it is often straightforward to determine what goes wrong with the protein as a result of a mutation. The HOPE^42^ software takes as input a protein sequence and a point mutation, and assumes that this mutation is causal for a genetic disorder. It then looks for the 3D structure of the protein, and when this one cannot be found, a homology model either will be extracted from the CMBI human protein homology model collection (HUMMOD) or will be constructed on the fly by YASARA_Model. HOPE takes the structure or homology model and calls nearly hundred web services from all over the internet. It then applies logic reasoning to determine the most probable cause for the relation between the protein mutation and the human disease state. HOPE is typically used 250 times per week.

HOPE uses a multiple sequence alignment too, mainly for an estimate of the variability. HSSP entries are available for all human protein sequences obtained from http://hgdownload.soe.ucsc.edu/goldenPath/hg19/chromosomes/. HUMMOD and these HG19‐HSSP entries are meant for use by bioinformaticians with good understanding of programming and MD5SUMs as MD5SUMs of the sequences are used as file names in both databases.

### 4.2 *HUMMOD holds all homology models that can be constructed for human proteins*

HUMMOD models are constructed conservatively, that is, the percentage sequence identity between template and model must be clearly above the threshold curve and must be longer than 20 residues. HUMMOD copes with situations in which the model is just one chain, but the template is a complex; in these cases, the chains needed to complete the model of the complex are looked up and modeled together with the requested chain. The HUMMOD database of human homology models is available at ftp://ftp.cmbi.umcn.nl/pub/molbio/data/hg-models/. Users can ask for the interactive modeling of missing files (https://www3.cmbi.umcn.nl/hommod/), or files with a different sequence than the one we premodeled through the server at ftp://ftp.cmbi.umcn.nl/pub/molbio/data/hg‐models/. The full HUMMOD modeling protocol will be made available in due time; the HUMMOD software pipeline is available upon request.

## 5 DISCUSSION

Databases come and go, but vanity and errors are for ever. Attwood et al. found in 2015 that more than 60 % of all databases that were in this century described in a publication in a refereed journal are no longer available, and half of all software in this field cannot be compiled easily by a computer expert.^43^ Many articles have been published on 10 simple rules for the design of (bioinformatics) software or databases. In one such article on community resources,^44^ one of us listed the 10 rules for making an internet‐based resource. These rules are all very broad and applicable to most systems that provide PDB‐wide precalculated data or any bioinformatics facility in general:

1. Longevity: The one rule to rule them all. Unless you can maintain your database for at least 10 years, then do not start.
2. Users: All databases need users and citations. To gain and keep users, you need to provide query and browsing interfaces as well as someone who answers emails.
3. Befriend Nucleic Acids Research and Database and similar journals: The descriptions of your database are essential to inform users. It is also essential to target publications to the readership.
4. Collaborate and be “open”: Your collaborators may offer an exit strategy in the future, and nobody is going to steal your resource.
5. Give credit: There is more than 100% to go around.
6. Automate: Too much manual intervention makes for an unsustainable database leading to premature death. You need to automate roughly 90% of everything, every year.
7. Do not invent a new standard. Use what exists.
8. Keep it simple: Google is a model interface.
9. Visibility: Be at the right conferences and be recognizable. Use the same logo and present a poster.
10. Exit strategy: At some point you will retire. Start planning early to ensure your database continues.

The retirement of the main author of the CMBI PDB facilities described in this article makes these 10 rules for internet‐based resources very timely. Some succession planning has been going on already: PDB‐REDO maintenance was taken over by the NKI in Amsterdam already 8 years ago, and DSSP is likely to follow next. BIPS is likely to take over the *Lists*. In due time, the main PDB‐facilities page at the CMBI is likely to point mainly or perhaps even only to sites outside the CMBI.

The journal *Nucleic Acids Research* (NAR) publishes an annual volume on databases, and on the web page http://www.oxfordjournals.org/nar/database/subcat/4/14, a list is provided of refereed databases that are one way or another related to protein structures. Entries in this list range from fully hand‐curated databases with just a few hundred entries like 2P2Idb^45^ that summarizes protein–protein interaction disruption examples to fully automatically PDB‐wide databases like PDBsum^36^ that summarizes PDB entries in a nicely human‐readable fashion; from catering for the bioinformatician like PDB‐REPRDB^46^ that provides culled data selections that are optimized for bioinformatics use to catering for the wider public like CATH^47^ that sorts proteins by fold; and from protein‐focused like DSSP to focused on ligands in PDB entries like ValidatorDB.^48^ Unfortunately, as was first made unambiguously clear by Pedro who produced in the early 1990s, the famous “Pedro's Research Tools” hotlist, web pages with tools and databases get outdated very quickly (see http://www.gen-info.osaka-u.ac.jp/pedro.html for an example). And indeed, even the curated and refereed NAR list of about a hundred protein structure‐related databases holds many dead links. Some databases stay alive even after the main author dies (e.g., the PMDB^49^ models database), but a sizeable fraction can only be found after some Googling or cannot be found at all. For most major bioinformatics databases, identifiers.org^50^ already provides unique namespace identifiers, as well as one or more locations of resources (“mirrors”) for the data (see https://registry.identifiers.org/registry/pdb for an example). We will attempt registering CMBI facilities here in over the next 2 years, because such curated database descriptions also provide a level of robustness against changing web addresses. The CMBI, for example, had to change its www address two times over the past 19 years due to university name changes. We see a role for a large player in the data field (Google or some international biodata‐related institute) to make available space for mirrors of published facilities. Such mirrors can then, for example, stay up for 5 years after the last update. ELIXIR might set up a committee to think about this topic; our evaluation of the NAR‐published facilities certainly hints at a necessity. On the other hand, sometimes a database is no longer needed. PDBsum, for example, was needed many years ago, but nowadays wwPDB provides almost all PDBsum information nearly as nicely as PDBsum. Consequently, the demise of PDBsum is now in the making (Roman Laskowsky, private communication). We do believe strongly, though, that the FAIR principles of data handling (https://en.wikipedia.org/wiki/FAIR_data) should be adhered to, independent of the data type, the data volume, or its perceived scientific value.

Almost all aspects of the CMBI PDB facilities are “open.” So, it is free to make copies, to make shadow/mirror web‐sites, and so on. However, we have spent close to a hundred man‐years producing and maintaining these facilities, and hope that people properly acknowledge where the data and software came from if they make copies.

## 6 THE FUTURE

The road to hell is paved with good intentions. Nevertheless, we have plans for extensions and improvements of the CMBI PDB facilities. These include small tweaks to the *Lists* databases. It can also be imagined that a series of *Lists* entries gets some extra information added at the end of the line like warnings for high B‐factors and partial occupancies, or for symmetry contacts. New *Lists* databases can be made easily and will, upon request, normally be made within a few weeks. HSSP entries presently are made for whole data entries. In the future, we will provide one HSSP entry per protein chain and add metadata that explains how to reconstruct whole HSSP entries from those. Additionally, mostly to save disk space but more importantly CPU time, we will no longer make frequent updates for HSSP entries that contain “enough” aligned sequences; how much is enough still has to be determined but this might become 5000 and include some function of their variability. At present, all macromolecular structures determined by NMR or electron microscopy are rejected from the *Lists* collection. We might at some time make PDB entries that contain for each NMR entry just one (the most representative) of the typical 20 copies, and use those as input for the *Lists* databases generator. We might also make separate database collections for low‐resolution PDB entries, for protein–nucleic acid complexes, for membrane proteins, and so on. In general, we are open for discussions about improvements and new PDB facilities.

## Footnotes

1 It is understandable that all these data are not publicly available as long as they have clear economic value to their producers, but it would be a pity if these would remain out of the public's eye indefinitely. We realize that depositing so much data would require substantial effort. Therefore, the authors of this article offer to help any industry that wants to upload large numbers of macromolecular structures to wwPDB.
